# Influence of altered torsional stiffness through sole modification of air pressure shoes on lower extremity biomechanical behaviour during side-step cutting maneuvers

**DOI:** 10.1371/journal.pone.0297592

**Published:** 2024-02-29

**Authors:** Md Samsul Arefin, Hsiao-Feng Chieh, Chien-Ju Lin, Cheng-Feng Lin, Fong-Chin Su

**Affiliations:** 1 Department of Biomedical Engineering, National Cheng Kung University, Tainan, Taiwan; 2 Medical Device Innovation Center, National Cheng Kung University, Tainan, Taiwan; 3 Department of Physical Therapy, College of Medicine, National Cheng Kung University, Tainan, Taiwan; 4 Department of Leather Engineering, Khulna University of Engineering & Technology, Khulna, Bangladesh; Ningbo University, CHINA

## Abstract

Directional changes in cutting maneuvers are critical in sports, where shoe torsional stiffness (STS) is an important factor. Shoes are designed based on different constructions and movement patterns. Hence, it is unclear how adjustable spacers into the sole constructions of air pressure chambers (APC) affect the STS in side-step cutting. Therefore, this study investigated the effects of altered STS through adjustable sole spacers on ground reaction force (GRF) and ankle and knee joint moments in side-step cutting. Seventeen healthy recreational athletes performed side-step cutting with experimental conditions including (i) barefoot (BF), (ii) unaltered shoes (UAS): soles consisting of APC, and (iii) altered shoes (AS): modified UAS by inserting elastomeric spacers into cavities formed by APC. Mechanical and biomechanical variables were measured. Significant differences were revealed across shoe conditions for impact peak (p = 0.009) and impulse (p = 0.018) in vertical GRF, time to achieve peak braking (p = 0.004), and peak propulsion (p = 0.025) for anterior-posterior GRF in ANOVA test. No significant differences were observed in GRF peaks and impulses between UAS and AS except for a trend of differences in impact peak (p = 0.087) for vertical GRF. At the ankle and knee joint, peak ankle power absorption (p = 0.019), peak knee internal rotation moment (p = 0.042), peak knee extension moment (p = 0.001), peak knee flexion moment (0.000), peak knee power absorption (p = 0.047) showed significant difference across three shoe conditions. However, no significant differences between the UAS and AS were noticed for peak joint moments and power. Altered shoe torsional stiffness did not significantly affect the peak forces and peak ankle and knee joint moments or powers; hence sole adjustment did not influence the cutting performance. This study might be insightful in sports footwear design, and adjusting shoe torsional stiffness by sole modification might be advantageous for athletes playing sports with cutting maneuvers to reduce the risk of injuries by controlling the twisting force at the ankle that frequently happens during cutting maneuvers.

## 1 Introduction

Sports maneuvers involving directional changes are critical during pivoting moments in sports and are connected to lower limb injuries [1]. Athletes perform a range of directional changes with different approaches, velocities, and angles during sports, and therefore, the capability of changing direction quickly and safely is critical [1]. A significant percentage of lateral cutting movements could depict over 50% of total movements noticed in specific team sports such as basketball, soccer, volleyball, and handball [2]. Athletes in a basketball game spent 31% of their playing time in directional movement change of the total movements, among which 20% are classified as high-intensity movements [3]. Knee and ankle injuries are the causes of 25–50% of all injuries and are associated with a significant proportion of rotational and lateral cutting movements in soccer and basketball [2, 4, 5]. Previous studies have noted that the side-step cutting maneuver is associated with a high risk of noncontact anterior cruciate ligament (ACL) injuries [6–8]. Likewise, landing maneuvers, particularly single-leg landings, tend to carry a high risk of noncontact ACL injuries [9, 10]. A 13-year study also reported the prevalence of noncontact ACL injuries in collegiate basketball and soccer players [11]. Lateral ankle sprains and instability caused by the center of pressure (COP) shifting are relatively frequent in side-step cutting [5, 12–14].

Side-step or lateral cutting generates rotational or twisting motion that exerts on the foot or shoe during shoe–ground contact [15, 16]. Twisting moments (TM) occur due to the shear forces generated between the foot and ground contact; hence, it can be described as the ground reaction force in response to torsional moments exerted by athletes about the perpendicular axis centered at the foot’s center of pressure (COP). It prevents the foot/shoe internal rotation from the body acceleration to change its direction [17, 18]. The TM is an indicator of lower limb torsional stress and is observed to be connected with tibial stress rupture in distance runners [19]. Different TM patterns affect lower limb kinematics and joint moments in the coronal and transverse planes, revealing a link with patellofemoral pain syndrome. At the same time, mechanical loads show potential for injury risk on lower extremity joints [20]. Hence, it is an important biomechanical parameter to determine essential clinical findings about the risk of injury and in the rehabilitation process [21, 22].

Evidence shows shoes with different sole and midsole constructions, for example, minimalist, maximalist, barefoot, ballet pointé, low-high heeled shoes, gradual increase in the lateral wedge hardness and variations in the midsole thickness affect the lower limb kinetics and kinematics related to athlete’s performance and the occurrence of injuries [23–28]. Recent research suggests that a bionic shoe with a barefoot outsole shape also alters human lower extremity biomechanics and reduces injury risk [27, 29]. Shoes composed of sliding structure—for instance, midsole having several distinct layers or hollow or depression in the outsole—were revealed to lessen the peak of the shear force and the time delay in the first peak of AP-GRF during walking [30, 31]. It is possible that cutting movements in different sports comprise a sharp turning or twisting of the body [32, 33]; therefore, the structural and material distortion of shoe construction in the horizontal and rotational directions may be highly likely to influence the sliding of outsole–ground surface and consequently affect the GRF [34]. So, lateral stability and the STS are significant in injury prevention [35].

Torsion of the foot can be specified as the relative rotation of the forefoot to the hindfoot in the coronal plane of movement [36], which causes a decrease in movement coupling between the two-foot segments [37]. The peak torsion angles generated to lessen the ankle inversion angle in barefoot conditions [2], which are found to decrease while wearing shoes [36]. Shoe torsional stiffness (STS) measures the resistance of twisting of a shoe about its longitudinal axis between the back part (heel) and the fore part (toe) [38]. Earlier studies about the STS evaluated the impact of the modifications of different shoe constructions, for instance, cushioned shoes designed with different upper and midsole with different material hardness [39], shoes with identical upper materials but one of which had a transverse cut 1 cm deep through the outsole material, while the other, the neutral shoe, had no changes [40], shoes with identical uppers but a modified outsole that partially covers the waist (midpart) part of the shoe [41], and shoes with same upper materials but modified stiffness using cuts and torsion bar in the sole [42]. These studies reveal no consistent findings in the literature about the impact of STS on the lower extremity kinetics and kinematics and their injury risk in sports. One study shows altered STS has apparent effects on foot kinematics [40], while another study observed no change in ankle kinematics and knee angular impulse [39]. An increase in STS resulted in augmented ankle eversion moments, which could raise the likelihood of ankle injuries while knee moments remain unaffected [41]. Nearly similar findings found that STS significantly impacts foot kinematics and joint loading at the ankle but not at the knee [42].

However, according to our current knowledge, there are no prior studies investigating the impact of the sole adjustment through the addition of spacers into different hollow cavity positions of air pressure chambers, especially on the GRF and twisting moments, ankle, and knee joint moments and power during a side-step cutting movement. It was assumed that modifying the hollow cavities in the sole construction by inserting vertical adjustable spacers might modify the shoe cushioning, which could alter the STS.

Consequently, this study aimed to investigate the immediate impact of sole adjustment using spacers on the GRF and contact time, which are associated with sports performance, twisting moments, and ankle & knee joint moments associated with injury risk during side-step cutting movement. It was hypothesized that an alteration of torsional stiffness might influence the peaks and impulses of the twisting moment and GRFs during the side-step cutting movements. It was also hypothesized that altered STS could alter ankle and knee joint moments and power.

## 2 Materials and methods

### 2.1 Participants

Seventeen male university participants (age 22.76 ± 2.63 years; height 173.06 ± 3.73 cm; body mass 64.69 ± 4.55 kg; body mass index 21.61 ± 1.55 kg/m²) took part in this research. Participants were devoid of injury or pain in their lower limbs during the testing period and fit any shoes with a size of US8, US9, or US10. Participants were involved in different sports (mostly basketball, badminton, volleyball, and ultimate frisbee) at least twice per week (2.53 ± 0.87 days/week). Before the experiments, each participant was wholly informed about the experimental procedures.

Research protocol and consent forms were approved by the Human Research Ethics Committee at National Cheng Kung University, Taiwan (Approval no. NCKU HREC-E-110–456-2). Participants provided informed consent in a written form and signed by each participant. The approved recruitment period for this study is November 18, 2021, to December 31, 2023.

### 2.2 Shoe conditions

**Fig 1:**
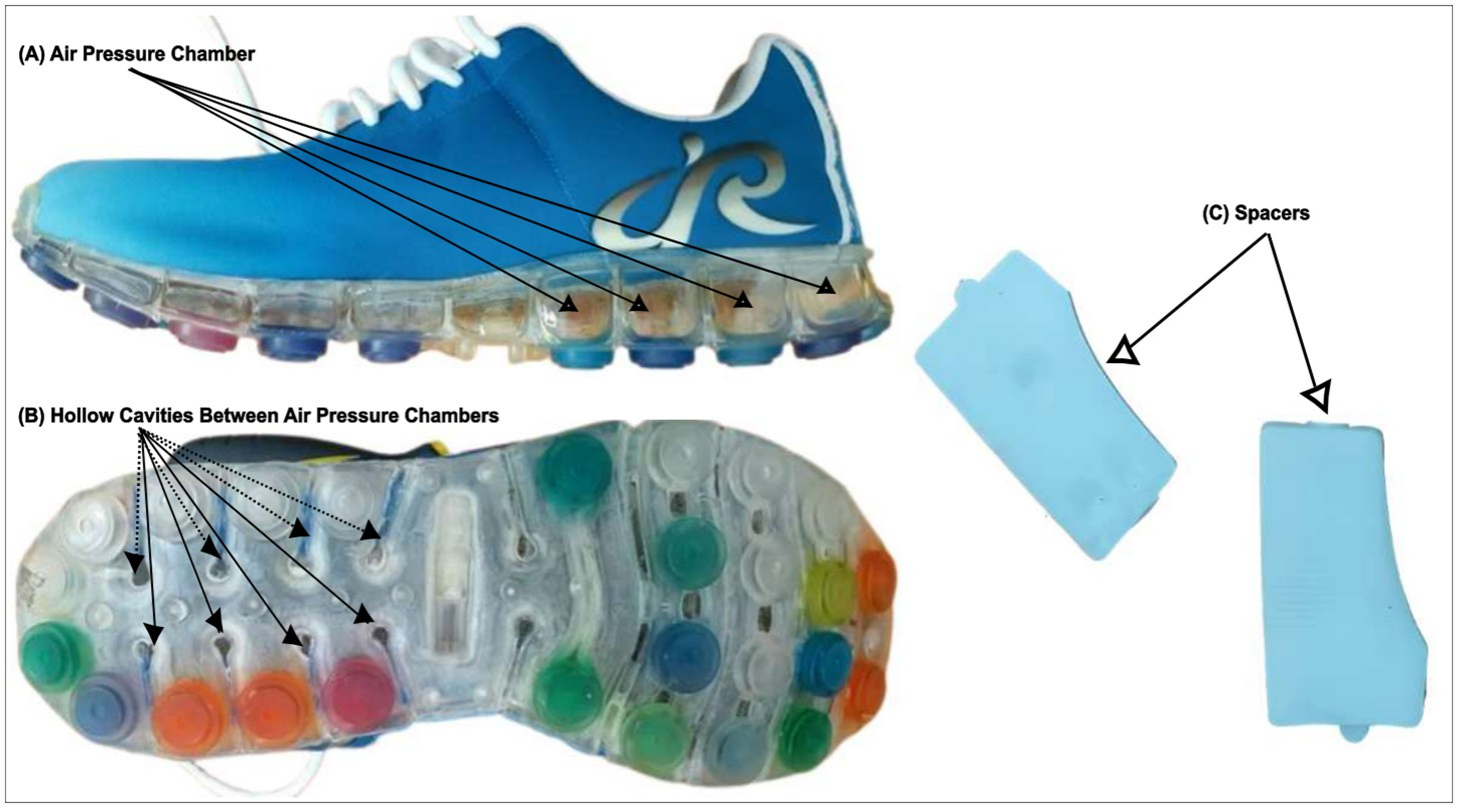
Photographs showing. (A) Air pressure chambers in the sole construction of the Dr. Air shoe, (B) Hollow cavities between air pressure chambers where spacers were inserted, and (C) Spacers.

This study investigated three experimental conditions: (i) barefoot (BF); (ii) unaltered shoe (UAS): air cushion shoe (Dr. aiR Inc., Taiwan) used without any modification; (iii) altered shoe (AS): air cushion shoe modified in the sole construction of the shoe using adjustable vertical spacers. Air cushion shoe (Fig 1A) is a running and walking shoe with a unique midsole construction consisting of several air chambers and hollow cavities (Fig 1B) between air pressure chambers. Air is injected into the chambers (hence called air pressure chamber) as shock-absorbing materials using a common air valve. This shoe is particularly designed for sole adjustment by adding and taking out vertical spacers (elastomeric and partially flexible material) in hollow cavities [43]. This sole construction is not conventional; generally, midsoles consisting of ethylene vinyl acetate and polyurethane foams [44] are used. A study noted that several factors, such as the forepart and backpart of shoe design, torsion elements, and even midsole and upper materials, can impact the shoe torsional stiffness [39]. For the altered shoe (AS), the sole construction was modified by adding eight spacers (Fig 1C) in the hollow cavities of air chambers positioned in the medial and lateral parts of the sole from the backpart to the midpart region of the sole with a uniform distribution. This study protocol included this modification to determine if any differences happened when testing the torsional stiffness and its effects on the cutting biomechanics.

It is essential to mention that 15 kPa of air (set by the manufacturer) was injected into the air pressure chamber of each shoe before each experiment to ensure the standard air pressure and that additional shoe lifts or orthotic insoles were not used in the shoes. All the shoes had identical uppers except for sole modifications, and the total weight of the shoe included the insole. Three different shoe sizes (US8, US9, and US10) were used (a total of 3 pairs of shoes) in this study to ensure a correct fit for all the participants.

### 2.3 Experimental set-up and test protocol

#### 2.3.1 Materials testing

**Fig 2:**
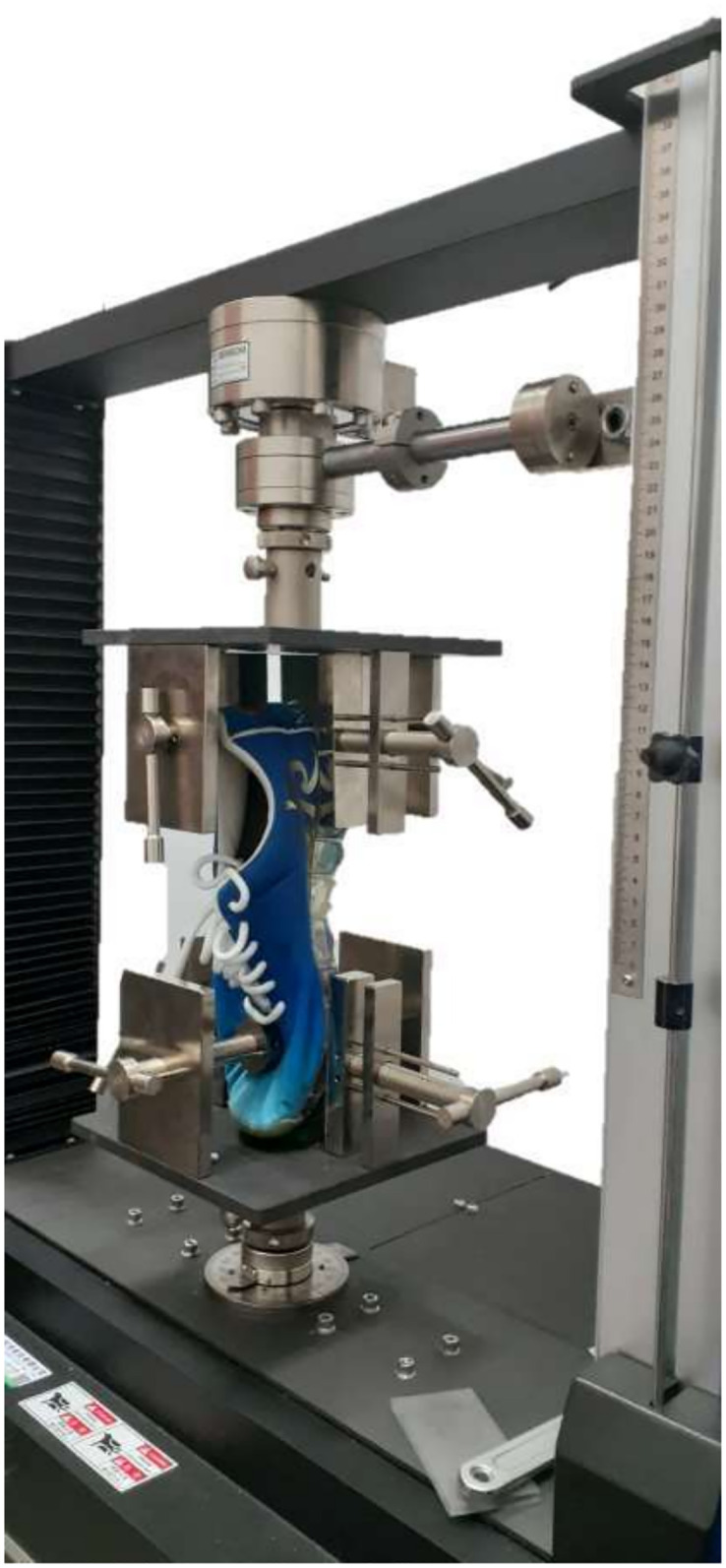
Materials testing system.

The torsional stiffness values of shoes were measured using a material testing machine (YM-T03/T07-Table Type, Yang Yi Technology Co. Ltd., Taiwan) that complied with the EN409 standard (Fig 2). Custom-designed fixtures were used to grip the shoe in the test machine. The upper fixture is attached to the moveable crossbeam having the loadcell, whereas the lower fixture was fixed to the lower crosshead of the test machine. The rear part of the shoe was gripped in the upper fixture, which was a stationary fixture. In contrast, the forepart of the shoe was fixed in the lower fixture, which was rotating/twisting about the long axis formed between the heel and toe parts of the shoe. The rotary head in the test machine is responsible for the rotation of the fixtures in a clockwise and anticlockwise direction that causes the shoe forepart movement against the stationary back part, and the movements are known as inversion and eversion of the shoe that could mimic the foot-shoe inversion/eversion motion. To measure the torsional stiffness of the shoe, the machine rotated the forefoot section of the shoe relative to the fixed rearfoot section with an angle of 30° of inversion and eversion, respectively, with angular speed of 1°/s [38, 39, 45] and sampling rate of 200 Hz [40].

Inversion and eversion torsion test data were collected in separate tests, and each test was repeated thirty times for unaltered (UAS) and altered shoes (AS) for each shoe size. Torsional moments, time stamps and torsion angles data were recorded during the test. Shoe torsional stiffness (Nm/°) was defined as the slope of the loading curve obtained from the torsional moments (Nm) versus the angle of twist (°) plotted on a graph. The curve fitting was conducted at the range between 5% and 25% of the maximum applied torsional moments for the loading curve using the least square method based on a previous study [46]. For each shoe condition, the first ten consecutive test cycles were excluded [47], and the average torsional stiffness was calculated from the 11th to 30th consecutive cycles (Table 1). The same researcher performed these tests for each shoe condition to ensure accurate measurements.

**Table 1:** Shoe torsional stiffness.

Shoe sizes	Inversional torsional stiffness (Nm/°)		Eversion torsional stiffness (Nm/°)	
	UAS Mean (SD)	AS Mean (SD)	UAS Mean (SD)	AS Mean (SD)
US8	0.115 (0.001)	0.128 (0.002)	0.105 (0.001)	0.147 (0.001)
US9	0.122 (0.001)	0.146 (0.001)	0.126 (0.001)	0.155 (0.002)
US10	0.135 (0.001)	0.177 (0.001)	0.133 (0.001)	0.146 (0.001)

Note: Torsional stiffness data are presented as mean ± standard deviations. The unit of torsional stiffness is Nm/°. Abbreviations: SD, standard deviation; UAS, unaltered shoe; AS, altered shoe; US, United States; Nm/°, Newton-meter per degree.

#### 2.3.2 Biomechanical test

At the beginning of the experiment, the participant’s demographic, and anthropometric information, including age, gender, body mass, height, and foot length, was documented. Based on the modified Helen Hayes marker system, 19 retroreflective markers [48] were placed bilaterally over the following anatomical landmarks of the subject’s lower extremity: the sacrum position, both anterior superior iliac spines (ASIS), the mid-anterior positions of both thighs, the medial and lateral epicondyles of both knees, the mid-anterior positions of both shanks, both ankle’s medial and lateral malleoli positions, center position between the second and third metatarsal bones of both feet, and both heels. For the shod conditions, markers were placed between the second and third metatarsal bones and heel markers were placed outside the shoe. Kinematic data were collected using an eight-camera, three-dimensional (3D) motion analysis system (Motion Analysis Corporation, Santa Rosa, USA) at a sampling frequency of 100 Hz. Ground reaction force data were recorded at 1000 Hz using one embedded force plate (9281B, Kistler, USA) in the motion track. During data collection, the cortex software synchronized the motion analysis system and force plate. Force plates were calibrated mandatorily before each trial by zeroing the force plate device. After marker placement, the participant was asked to stand upright in the center of the calibration area with lower and upper limbs staying neutral, palms facing forward, and correct head position with straight eyes to collect a static calibration trial. Once the calibration trial was captured, left and right limb ankle and knee medial markers (a total of four markers) were removed before starting the dynamic trials. Before the beginning of dynamic trials, the experimental procedures were demonstrated to the participants. Participants were instructed to practice side-step cutting on the 7-meter laboratory walkway at a self-paced speed [49, 50] as fast as possible with each experimental condition until they became familiar with the test procedure, including cutting speed and shoe conditions.

**Fig 3:**
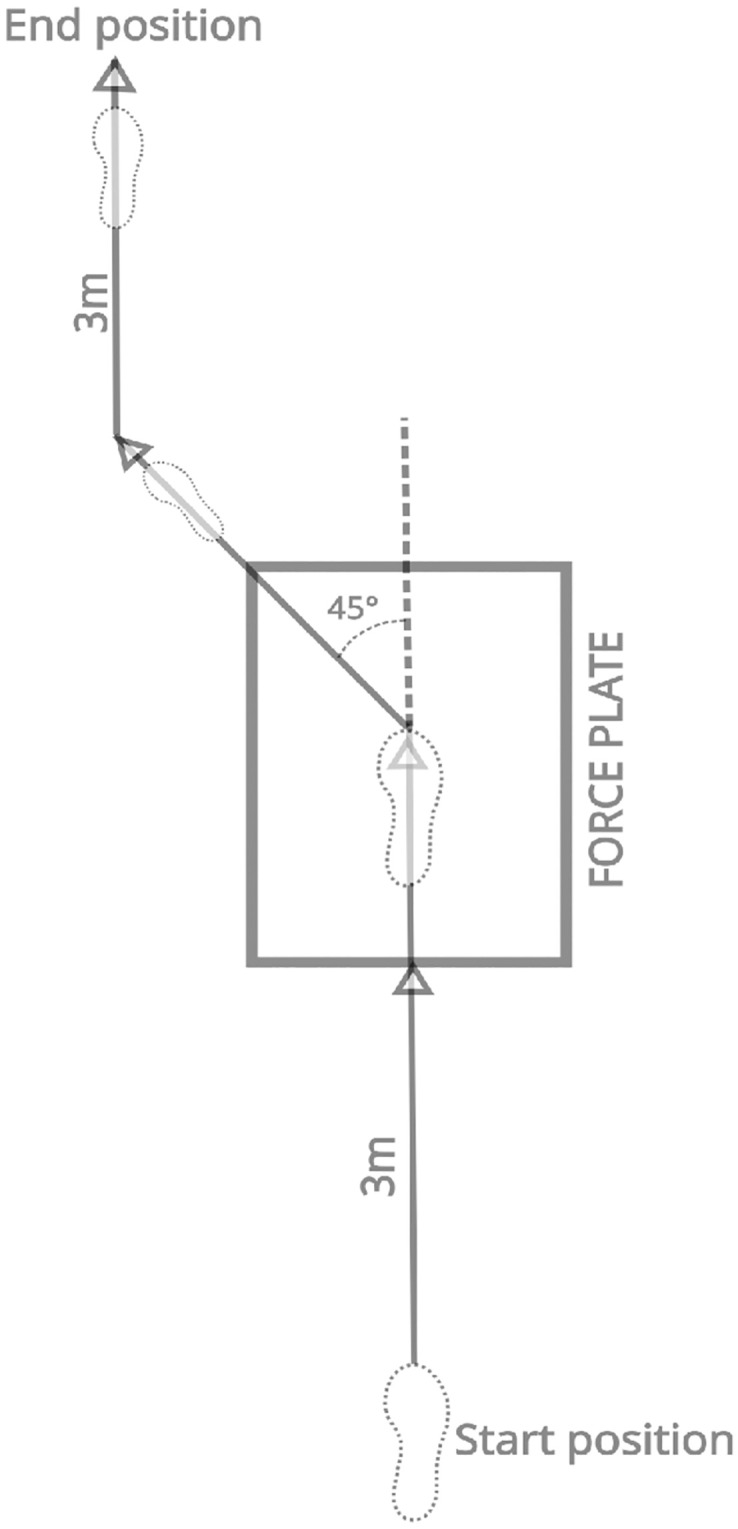
Diagram of side-step cutting movement.

In the side-step cutting protocol (Fig 3), participants started running toward the force plate at their self-selected speed from a standing position three meters behind it to confirm that the desired self-selected speed was obtained. When they reached the force plate, they used their right leg to move onto the force plate and, soon after that, ran away from the plate using the left leg with a 45° cutting angle in the left direction. After leaving the force plate, running continued to 3 meters to reach the end position. A modified movement pattern was adapted from a previous study [41]. There was an indication in the walkway for the starting position, the position to start the cutting movement (direction change) after the contact on the force plate, the direction to leave the force plate, and finally, the running direction of the remaining parts in the walkway. Trials were deemed successful when the participant placed the designated foot entirely on the force plate. Each participant performed trials with every shoe condition randomly and performed at least five trials for each condition. Participants were given a rest period of one minute between each trial and five minutes between each alteration of shoe condition [51, 52]. After a participant completed five successful trials in one shoe condition, the same procedure was repeated for the following two shoe conditions. Marker attachment and sole adjustment were done for every participant by the same experienced individual to ensure the experiment’s reliability. The three best trials were selected for each shoe condition for each participant for data analysis [51, 53]. Trials were excluded if participants’ entire feet did not come into contact with the force plate or if any markers were lost during the experiment.

### 2.4 Data reduction and analysis

The best three trials of motion capture data, where the participant successfully hit the force plate, were selected for analysis. Marker trajectories were identified using Cortex 8 motion analysis software and were smoothed using a low-pass, fourth-order, Butterworth filter with an 8 Hz cutoff frequency, while ground reaction force data were filtered with a fourth-order Butterworth 35 and 50 Hz low-pass filter [54–56]). Joint kinetic variables (ankle and knee joint moments and power) were calculated based on the Helen Hayes biomechanical model used in the Orthotrack ® 6.6.1 software (Motion Analysis Corporation, Santa Rosa, CA, USA) [57]. Torsional stiffness, ground reaction force and twisting moment variables were calculated using MATLAB custom-written codes (R2022a, The MathWorks Inc, USA). Vertical GRF variables including impact peak, active peak, impulse, the timing of impact and active peak of vertical GRF; variables of medio-lateral GRF such as force peak, impulse peak, and timing to peak, and anterior-posterior GRF variables including peak force, impulse and timing of both braking and propulsion were analyzed in this study and these variables have a direct relationship with side-step cutting [30, 51, 58]. This study also evaluates the twisting moment (also called free moment), ankle and knee joint peak moments, and power variables as they have a significant association with these parameters [17, 19, 20, 51, 59].

The gait cycle was defined as starting with the right foot heel strike on the force plate and concluding with successive heel strikes of the same foot. The first heel strike was identified using the analog graph of force plate data, and the second heel strike was determined using the right heel marker trajectory data in cortex 8 motion analysis software [60, 61]). The anterior-posterior ground reaction force was divided into braking and propulsive components. Braking force represented the ground pushing the participant posteriorly, while propulsive force represented the ground pushing the participant anteriorly. The braking and propulsion forces of AP-GRF were determined based on the concept [60, 62]. Impulse was calculated as the product of the area under the GRF curve and 1/sampling frequency based on the collected data, then normalized to the body weight in kg [63]. The twisting moment (TM) of ground reaction was calculated using equations that define the moment and force components in the force plates based on the manufacturer’s guidelines (Kistler Instrument AG, Winterthur, Switzerland). In this study, TM was calculated considering right leg stance phases on the force plate during side-step cutting and specified as positive in the adduction direction.

TM=Mz-Fy*ax+Fx*ay

Where Mz is the plate moment about Z-axis (vertical axis) in the force plate center, Fx is the GRF in X-direction (medio-lateral axis and positive direction to the right), Fy is the GRF in Y-direction (anterior–posterior axis, running direction is positive to Y-axis), ax is the X-coordinate of force application point (COP), and ay is Y-coordinate of force application point (COP) [64]. GRF and ankle and knee joint moments data were normalized to body weight (BW) [51], while the twisting moment was normalized to the product of body weight (BW) and body height (BH) multiplied by 10^-3^ [65]. The kinematic and kinetic parameters of the ankle and knee joints and GRF variables were time-normalized from 0 to 100% of the gait cycle (GC) [66]). The time taken for the impact peak and active peak of vertical GRF were measured from heel strike until the first and second GRF peaks, respectively, were detected. Likewise, the time to braking peak and propulsion peak of anterior-posterior GRF was defined as starting from heel strike until negative and positive peaks of anterior-posterior GRF, respectively, were calculated. Mean values were computed for each variable across all participants within each shoe condition.

### 2.5 Statistical analysis

Statistical analyses were conducted with SPSS software (Version 17, IBM, USA). The average of three trials for each variable was calculated for each participant for statistical analysis. The Shapiro–Wilk test was conducted to evaluate the normality of the dataset. Biomechanical variables were normally distributed except for the initial peak twisting moment and ankle external rotation moment. Regarding biomechanical variables, one-way repeated measures ANOVA test was conducted for normally distributed datasets to compare three shoe conditions as a repeated measure (within-subject factors) for each dependent variable [67]. Friedman test was used for the datasets with non-normal distribution. Greenhouse-Geisser’s epsilon adjustment was applied in the ANOVA test for all the variables when Mauchly’s test showed a violation of the sphericity assumption. When significant effects were observed in the ANOVA test, Fisher’s least significant difference (LSD) post hoc test was conducted [68]. The statistical significance level was set as p<0.05.

ANOVA test found a significant difference when comparing the variable, the impact peak of vertical GRF among different shoe conditions (BF, UAS, and AS) using repeated measures ANOVA within-subject factors (partial eta-squared = 0.20). A post hoc power analysis with G*power software [69]) showed a robust statistical power of around 99%, indicating this study’s ability to detect significant differences if the partial eta-squared value of 0.20, the significance level p = 0.05, and 17 participants were included [70]).

## 3 Results

Table 1 shows the mean and standard deviation (SD) of shoe torsional stiffness quantified by the material testing system for the UAS and AS conditions for both inversion and eversion movements. Changes in shoe torsional stiffness were observed for the altered shoe conditions. Table 2 displays the mean and standard deviation (SD) of peak GRF and TM variables for three shoe conditions. Table 3 shows corresponding data of peak joint moments and power variables for ankle and knee joints.

**Table 2:** Peak ground reaction forces and twisting moments variables for shoe conditions.

Biomechanical parameters	BF Mean (SD)	UAS Mean (SD)	AS Mean (SD)	*p-value*
**Time**				
Contact time (s)	0.251 (0.036)	0.252 (0.037)	0.251 (0.037)	0.886
**Twisting Moment (TM)**				
First Peak TM (% BW x BH x 10^-3^)	9.187 (8.100)	10.220 (7.956)	9.483 (6.975)	0.662
Second Peak TM (% BW x BH x 10^-3^)	7.363 (4.934)	7.854 (4.680)	7.140 (3.714)	0.714
Impulse TM (BWms)	0.190 (0.114)	0.202 (0.103)	0.187 (0.091)	0.748
**Vertical GRF**				
Impact Peak VRT-GRF (BW)	1.209 (0.352)*^†^	1.369 (0.374)	1.424 (0.374)	**0.009**
Active Peak VRT-GRF (BW)	1.947 (0.297)^†^	2.020 (0.358)	2.035 (0.346)	0.054
Impulse VRT-GRF (BWs)	0.300 (0.043)*^†^	0.313 (0.051)	0.313 (0.045)	**0.018**
Timing Impact Peak VRT-GRF (%GC)	5.278 (2.608)	6.163 (0.861)	5.841 (0.814)	0.245
Timing Active Peak VRT-GRF (%GC)	17.720 (2.441)	19.047 (2.331)	18.673 (3.015)	0.070
**Medio-Lateral GRF**				
Peak ML-GRF (BW)	0.324 (0.106)	0.355 (0.071)	0.358 (0.081)	0.230
Impulse ML-GRF (BWs)	0.050 (0.016)	0.053 (0.017)	0.053 (0.016)	0.331
Timing Peak ML-GRF (%GC)	19.439 (3.174)	20.343 (4.001)	19.266 (4.261)	0.472
**Anterior-Posterior GRF**				
Peak Braking AP-GRF (BW)	-0.333 (0.087)	-0.342 (0.071)	-0.344 (0.019)	0.739
Peak Propulsion AP-GRF (BW)	0.240 (0.050)	0.227 (0.054)	0.215 (0.042)	0.091
Impulse Braking AP-GRF (BWs)	-0.024 (0.008)	-0.023 (0.006)	-0.024 (0.009)	0.757
Impulse Propulsion AP-GRF (BWs)	0.017 (0.005)	0.016 (0.005)	0.015 (0.004)	0.225
Timing Peak Braking AP-GRF (%GC)	10.097 (2.158)	11.188 (1.087)	11.238 (1.529)	**0.004**
Timing Peak Propulsion AP-GRF (%GC)	30.889 (1.100)	32.413 (4.145)	32.232 (4.312)	**0.025**

Note: Data are presented as mean and standard deviation (SD). Positive indicates propulsion forces, while negative indicates braking forces.

* indicates significant difference from the UAS and

^†^ indicates significant difference from the AS. Bold p-value indicates significant difference in the repeated measures ANOVA test and multiple pairwise comparisons were done by the post hoc test Fisher’s least significant difference (LSD) method. The significance level was p<0.05. Abbreviations: BF, barefoot; UAS, unaltered shoes; AS, altered shoes; s, second; BW, body weight; BH, body height; BWms, body weight meter second; BWs, body weight second; %GC, percentage of gait cycle.

**Table 3:** Variables for peak ankle and knee joint moments and power for shoe conditions.

Biomechanical parameters	BF Mean (SD)	UAS Mean (SD)	AS Mean (SD)	***p-value***
**Ankle Joint Moment and Power**				
Peak Ankle Plantar Flexion Moment (Nm/kg)	1.949 (0.540)	1.780 (0.337)	1.778 (0.383)	0.180
Peak Ankle Eversion Moment (Nm/kg)	0.476 (0.273)	0.589 (0.213)	0.575 (0.265)	0.076
Peak Ankle External Rotation Moment (Nm/kg)	0.116 (0.100)	0.076 (0.072)	0.082 (0.073)	0.559
Peak Ankle Internal Rotation Moment (Nm/kg)	-0.096 (079)	-0.100 (0.065)	-0.095 (0.075)	0.083
Peak Ankle Power Absorption (W/kg)	-3.356 (1.470)*^†^	-2.237 (1.153)	-2.098 (1.091)	**0.019**
Peak Ankle Power Generation (W/kg)	7.238 (2.557)	7.196 (2.017)	7.015 (2.070)	0.824
**Knee Joint Moment and Power**				
Peak Knee External Rotation Moment (Nm/kg)	0.053 (0.065)	0.039 (0.045)	0.045 (0.068)	0.325
Peak Knee Internal Rotation Moment (Nm/kg)	-0.275 (0.173)^†^	-0.320 (0.134)	-0.326 (0.149)	**0.042**
Peak Knee Abduction Moment (Nm/kg)	0.463 (0.346)	0.597 (0.479)	0.598 (0.515)	0.101
Peak Knee Adduction Moment (Nm/kg)	-0.219 (0.214)	-0.159 (0.146)	-0.177 (0.167)	0.113
Peak Knee Extension Moment (Nm/kg)	2.424 (0.508)*^†^	2.772 (0.455)	2.768 (0.485)	**0.001**
Peak Knee Flexion Moment (Nm/kg)	-0.389 (0.063)*^†^	-0.343 (0.054)	-0.351 (0.055)	**0.000**
Peak Knee Power Generation (W/kg)	4.442 (1.278)	4.681 (1.495)	4.866 (1.379)	0.441
Peak Knee Power Absorption (W/kg)	-8.336 (3.365)^†^	-9.890 (3.546)	-9.644 (3.413)	**0.047**

Note: Data are presented as mean and standard deviation (SD). Positive indicates plantar flexion, eversion, external rotation, abduction extension moments, and power generation. Negative indicates internal rotation, adduction, flexion moment, and power absorption.

* indicates significant difference from the UAS and

^†^ indicates significant difference from the AS. Bold p-value indicates significant difference in the repeated measures ANOVA test and multiple pairwise comparisons were done by the post hoc test Fisher’s least significant difference (LSD) method. The significance level was p<0.05. Abbreviations: BF, barefoot; UAS, unaltered shoes; AS, altered shoes; Nm/kg, Newton-meter per kilogram; W/kg, watts per kilogram.

### 3.1 Contact time, and peaks of ground reaction forces and impulses

**Fig 4:**
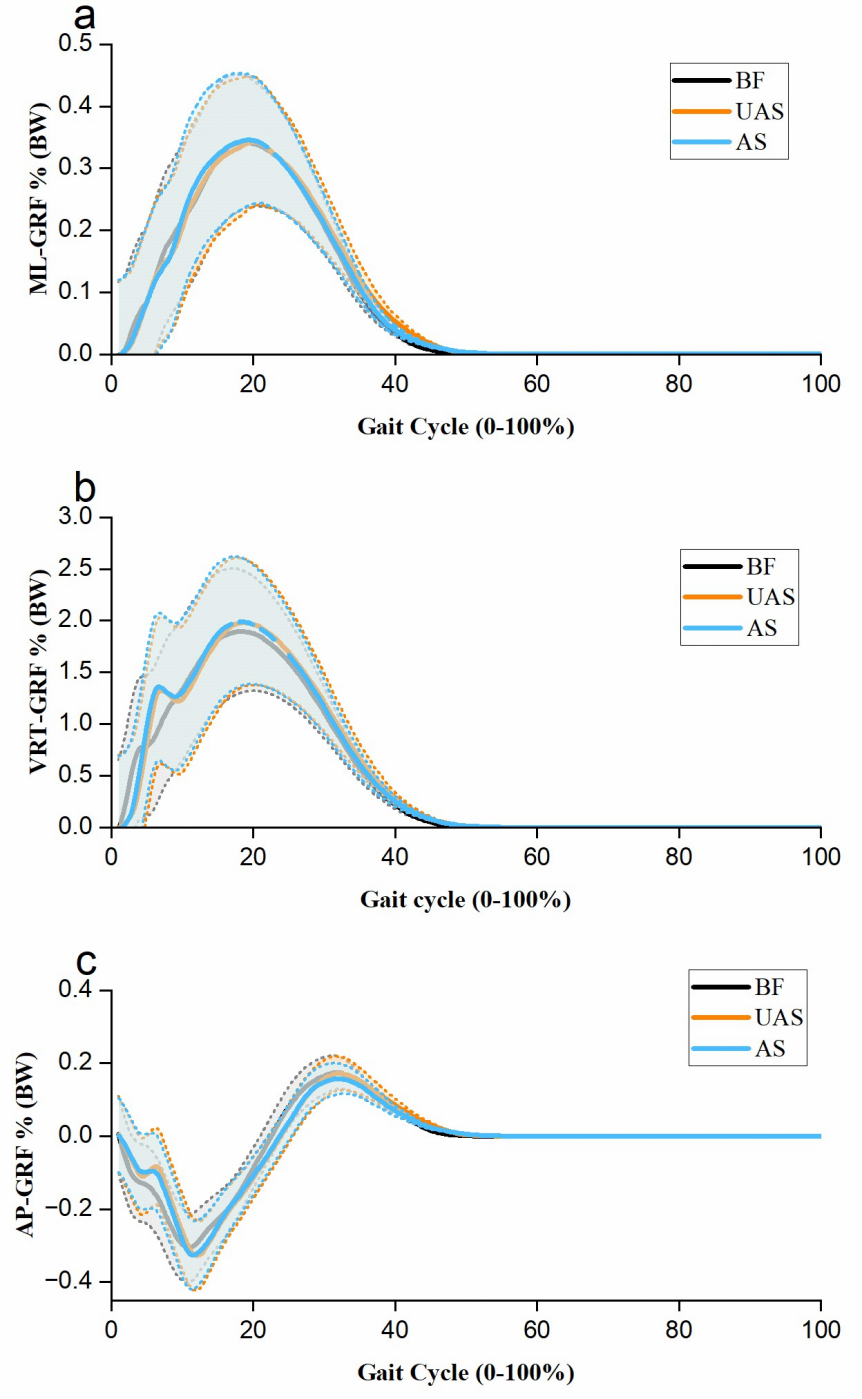
Ground reaction force components for experimental conditions. Fig 4a shows mean medio-lateral ground reaction force (ML-GRF curves, Fig 4b shows mean vertical ground reaction force (VRT-GRF) curves, and Fig 4c shows mean anterior-posterior ground reaction force (AP-GRF) curves with shade for the barefoot (BF), unaltered shoe (UAS) and altered shoe (AS) during the gait cycle. The shading is shown as one standard deviation (± 1 STD).

No significant difference was noted in contact time (F (1.498, 23.970) = 0.069, p = 0.886) across the different shoe conditions. The mean GRF curves were presented in Fig 4 for three experimental conditions (BF, UAS, and AS) during the gait cycle. For VRT-GRF, AS showed a significantly higher impact peak (p = 0.007), active peak (p = 0.027), and impulse (p = 0.035) compared with BF. Similarly, UAS observed a significantly larger impact peak (p = 0.028) and impulse (p = 0.020) compared with BF, except for the active peak (p = 0.104) in VRT-GRF. Although no significant difference was observed, a trend of differences in impact peak in VRT-GRF (p = 0.087) was detected between AS and UAS shoe conditions.

For ML-GRF, shoe conditions did not show a significant difference in the ANOVA test for any of the peak force (F (1.240, 19.842) = 1.568, p = 0.230), impulse (F (2, 32) = 1.131, p = 0.331), or time to achieve peak force (F (2,32) = 0.768, p = 0.472). For AP-GRF, AS revealed a significant difference in time to achieve peak braking forces (p = 0.001) and peak propulsion force (p = 0.037) compared with BF. Similarly, UAS observed a significant difference in time to achieve peak braking forces (p = 0.015) and peak propulsion force (p = 0.039) compared with BF. However, no significant differences were noted between AS and UAS for either peak braking or propulsion forces.

### 3.2 Peaks and impulses of twisting moments

**Fig 5:**
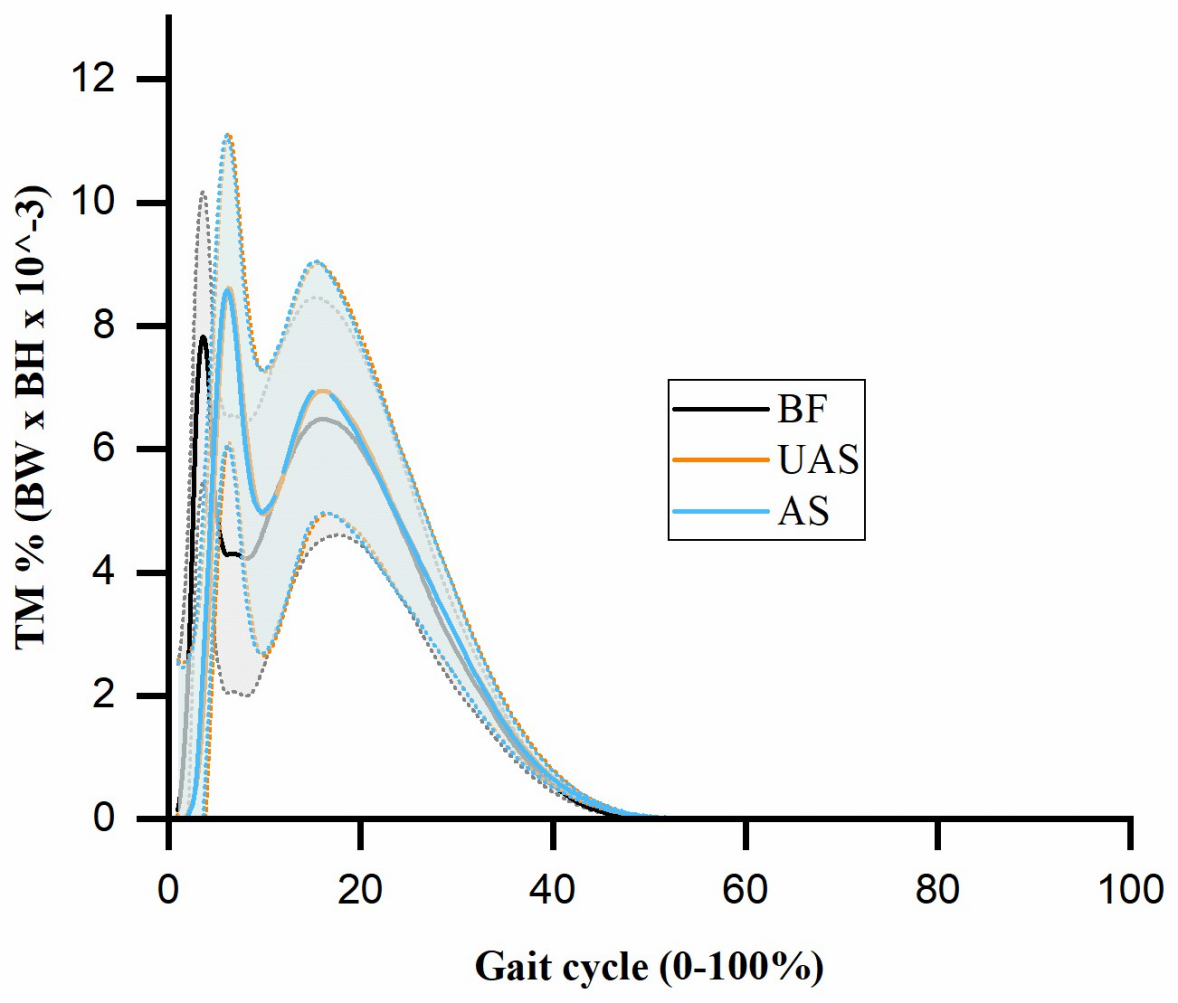
Mean twisting moments (TM) curves with shade for barefoot (BF), unaltered shoe (UAS), and altered shoe (AS) during the gait cycle. The shading is shown as one standard deviation (± 1 STD).

The mean twisting moments curves were presented in Fig 5 for three experimental conditions (BF, UAS, and AS) during the gait cycle. The twisting moments were normalized to the product of body weight (BW) and body height (BH) multiplied by 10^-3^. No significant difference was found in the ANOVA test for the first peak (p = 0.662), second peak (p = 0.714), or impulse (p = 0.748) of the twisting moment across three shoe conditions.

### 3.3 Ankle peak joint moments and power

No significant difference was revealed in the joint moment variables, including peak plantar flexion moment (F (1.196, 19.143) = 1.931, p = 0.180), peak eversion moment (F (1.459, 23.346) = 3.122, p = 0.076), peak external rotation moment (p = 0.559), or peak internal rotation moment (F (1.474, 23.589) = 0.083, p = 0.083) among the three shoe conditions. However, no significant difference was found, a trend indicating differences in the peak values of the eversion moment (p = 0.076) and internal rotation moment (p = 0.083). The AS revealed a significantly smaller magnitude of peak power absorption (p = 0.008) compared with BF for the joint power. Similarly, UAS observed a significantly lower magnitude of peak power absorption (p = 0.042) compared to BF.

### 3.4 Knee peak joint moments and power

The AS observed a significantly higher peak knee internal rotation moment (F (1.387, 22.194) = 4.169, p = 0.018) in the transverse plane and a higher peak knee extension moment (F (1.421, 22.742) = 10.958, p = 0.000) and lower flexion moment (F (2, 32) = 16.195, p = 0.000) in sagittal motion plane compared to BF. Similarly, UAS noted a significantly larger peak knee extension moment (F (1.421, 22.742) = 10.958, p = 0.006 and lower flexion moment (F (2, 32) = 16.195, p = 0.000) in sagittal motion plane compared to BF. However, no significant difference was detected between AS and UAS for knee joint moment variables. For knee joint power, higher knee peak power absorption (F (1.388, 22.205) = 3.981, p = 0.014) in AS was observed compared with BF. Analysis of foot marker data revealed the maximum angular velocity of foot inversion, and eversion was nearly 200°/s for the cutting movement.

## 4 Discussion

This study investigated the immediate effects of incorporating spacers into the cavity of sole construction having air pressure chambers during side-step cutting motion. It was hypothesized that inserting vertical spacers into the hollow cavities of the sole construction would modify the stiffness of the air cushioning of the shoe. Hence, this modification might affect the STS. This study aimed to evaluate the impact of the altered STS on various factors related to sports performance, such as ground reaction forces (GRF) and contact time. Additionally, the investigation focused on assessing the impact of twisting moments and the joint moments and powers at the ankle and knee, which have been linked to injury risk.

Contrary to our hypotheses, results showed that GRF variables increased, such as impact peak, active peak, and impulse. At the same time, ankle power absorption decreased, possibly due to the shoe effect for AS and UAS compared to BF. However, no noteworthy differences were found in these variables when comparing the AS and UAS. Even though differences were not significant, a trend of differences was observed in the VRT-GRF impact peak (p = 0.087) between AS and UAS and in the active peak between BF and AS, which might be achieved due to the outsole adjustment that causes altered shoe torsional stiffness. This result aligns with a previous study demonstrating a notable difference in active and propulsive VRT-GRF peaks between barefoot and open-cut footwear [42]. Nevertheless, no differences were observed in the other peak GRFs when comparing the fixed, open, and open-cut shoe conditions that featured identical shoe uppers and a unique torsion bar component, where the fixed shoe torsion bar could not rotate due to locking and the open and open-cut shoes had unrestricted rotation in the same study [42]. According to another study, a higher active peak GRF may not always lead to a greater impulse or a reduced time in the stance phase for optimal athletic performance [59]. As shown by previous research, the likelihood of experiencing overuse injuries may be higher due to increased peak joint moments caused by significant active peak GRF [51]. These findings align with the observations revealed in the current study when comparing BF and AS.

This study noted no significant differences in the impact peak of VRT-GRF and the peak braking or propulsion force of AP-GRF. These findings were similar to a prior study that revealed the impact force’s magnitude was unaffected by shoe cushioning properties [71]. This implies that the study participants modified their running technique to accommodate different shoe conditions [71]. It should be noted that reducing the impact peak of VRT-GRF has been a crucial issue for shoe producers and designers [30]. Shoe conditions in the present study did not change medio-lateral shear impulse or contact time, implying that sole adjustment might not alter side-step cutting performances. A previous study noted the same findings and revealed that using shear-reduction footwear did not affect the shear impulse generated in the propulsion or the timing of contraction during side-step cutting, thus implying no alteration in performance [51]. In the present study, shoe conditions showed significant differences in timing to achieve peak magnitudes for braking (p = 0.004) and propulsion forces (p = 0.025) among three shoe conditions. Although not statistically significant, there was a tendency of differences in timing to obtain peak magnitude for VRT-GRF impact (p = 0.087) between AS and UAS. Evidence showed similar results explained the time to achieve first peak AP-GRF delayed by a straight groove-type shear cushioning shoe during walking [30]. A reduced timing to reach peak impact indicates a greater loading rate associated with a higher possibility of sports injuries [30, 72, 73]. Contrary to our expectation in this study, the TM revealed no significant differences in its first peak, second peak, and impulses across shoe conditions for the side-step cutting movement. A study supported these findings noted the absence of any significant difference between the shear reduction shoe and the control shoe during cutting movement [51].

At the ankle joints, this study did not notice significant differences in joint loadings, including peak moments in internal/external rotation, eversion, or plantar flexion, across all three planes of motion. However, a tendency of differences was revealed in peak internal rotation (p = 0.083) and eversion moment (p = 0.076). Identical results disclosed in an earlier study showed no differences in loading in the frontal plane for different shoe conditions except for the other two planes of movement [42]. Shoe conditions AS and UAS showed significantly lower peak ankle power absorption compared to BF, but no notable difference was found between AS and UAS. This result partially contrasts with a study in which peak negative (absorption) power in the sagittal motion plane was increased for neutral and anti-pronation shoes [74]. Any changes in the foot kinematics due to sole modification that altered torsional stiffness might be compensated at the ankle joint; however, compensation might not happen because differences in the ankle joint moments are insignificant for the shoe conditions.

At the knee joints, significant differences were measured solely when AS and UAS compared to BF in the transverse motion plane for peak internal rotation moment but for peak extension and flexion moments in the sagittal plane of motion. However, no joint moment variables at the knee joint observed differences between AS and UAS. In this study, knee joint moments were not affected due to changes in torsional stiffness through sole modification, which is a significant finding, because runners often suffer from knee joint injuries, and a prior investigation found similar results [41]. The AS showed decreased knee peak power absorption compared to the BF, but no difference was noted in knee power variables between AS and UAS.

### 4.1 Limitations and future studies

Recognizing the current study’s limitations is important while analyzing the results. First, only male university recreational athletes participated in this study, and therefore, it may not be appropriate to generalize the findings to female and professional athletes. Then, the shoe sample used in this study was commercially accessible and had various shoe constructions; hence, the findings might not be conclusive for all shoe models. Moreover, the current study did not evaluate the long-term effects of sole modification. It did not compare it with a traditional-cushion shoe, so future studies are needed to verify these results. Fourteen participants had dominant right legs, and the fact that the other three had dominant left legs could be seen as an additional limitation of this study. Finally, foot and ankle movements were analyzed as the markers were attached to the shoe upper, which might lead to data analysis errors. Therefore, more studies might be necessary to explore more insights about shoe torsional stiffness, considering shoe design perspectives for athletes’ performance and risk factors of lower limb injuries.

## 5 Conclusions

This study examined how the modified STS, through sole adjustment, alters lower limb biomechanics during side-step cutting maneuvers. It was assumed that the sole modification might play an important role in altering the STS, which in turn would affect the cutting mechanics. The results, however, did not show significant differences in the impact peak, active peak, or impulse of VRT-GRF between UAS and AS but observed significant differences between BF and AS and between BF and UAS. No significant difference was noticed in the impact peak of VRT-GRF, the braking peak and propulsion peak of AP-GRF, or the contact time across shoe conditions, indicating no alteration in cutting performance. Altering the time to achieve peak braking and peak propulsion force in the AP direction implied altering the loading rate, which has been linked to the risk of injury in sports. Altered STS did not affect significantly joint moments or powers in the sagittal motion plane at the ankle or knee joints and, consequently, does not influence the cutting performance. This study might provide insights and potentially guide future sports footwear design, focusing on controlling the twisting force exerted, particularly on the ankle joint, during cutting maneuvers and minimizing the risk of injury.

## Supporting information

S1 Data Biomechanical and materials test dataset. The S1 data file contains 17 participant’s biomechanical test data averaged for each participant for three experimental conditions and normalized to the percentage of gait cycle. Biomechanical test variables include ground reaction force (GRF) components, twisting moments (TM), ankle joint moments and powers (AJMP), and knee joint moments and powers (KJMP). Materials test variable includes torsional stiffness (TS). Each variable’s data is provided as separate xlsx files.
(ZIP)

## Abbreviations

AP: Anterior-Posterior
APC: Air Pressure chamber
APS: Air Pressure Shoe
AS: Altered Shoe
BH: Body Height
BW: Body Weight
COP: Center of Pressure
GC: Gait Cycle
GRF: Ground Reaction Force
ML: Medio-Lateral
STS: Shoe Torsional Stiffness
TM: Twisting Moments
UAS: Unaltered Shoe
VRT: Vertical
