# Supplementary material for: Influence of altered torsional stiffness through sole modification of air pressure shoes on lower extremity biomechanical behaviour during side-step cutting maneuvers
Source: PLoS One. 2024 Feb 29;19(2):e0297592. doi: 10.1371/journal.pone.0297592 (PMC10903810; doi:10.1371/journal.pone.0297592)
Supplement: S1 Data — The S1 data file contains 17 participant’s biomechanical test data averaged for each participant for three experimental conditions and normalized to the percentage of gait cycle. Biomechanical test variables include ground reaction force (GRF) components, twisting moments (TM), ankle joint moments and powers (AJMP), and knee joint moments and powers (KJMP). Materials test variable includes torsional stiffness (TS). Each variable’s data is provided as separate xlsx files. (ZIP) [file pone.0297592.s001.zip › S1 data/README.docx]

**Dataset Description**

**Title:** Raw Dataset as a supplement to the article: Influence of Altered Torsional Stiffness Through Sole Modification of Air Pressure Shoes on Lower Extremity Biomechanical Behaviour During Side-step Cutting Maneuvers

**Data Dictionary:**

| **Variable Name** | **Abbreviation** | **Description (Unit)** |
| --- | --- | --- |
| **Ground Reaction Force (GRF)** | | |
| Barefoot Vertical Ground Reaction Force | BF-VRT-GRF | %BW |
| Unaltered Shoe Vertical Ground Reaction Force | UAS-VRT-GRF | %BW |
| Altered Shoe Vertical Ground Reaction Force | AS-VRT-GRF | %BW |
| Barefoot Anterior-Posterior Ground Reaction Force | BF-AP-GRF | %BW |
| Unaltered Shoe Anterior-Posterior Ground Reaction Force | UAS-AP-GRF | %BW |
| Altered Shoe Anterior-Posterior Ground Reaction Force | AS-AP-GRF | %BW |
| Barefoot Medio-Lateral Ground Reaction Force | BF-ML-GRF | %BW |
| Unaltered Shoe Medio-Lateral Ground Reaction Force | UAS-ML-GRF | %BW |
| Altered Shoe Medio-Lateral Ground Reaction Force | AS-ML-GRF | %BW |
| **Twisting Moment (TM)** | | |
| Barefoot Twisting Moment | BF-TM | % BW x BH x 10-3 |
| Unaltered Shoe Twisting Moment | UAS-TM | % BW x BH x 10-3 |
| Altered Shoe Twisting Moment | AS-TM | % BW x BH x 10-3 |
| **Ankle Joint Moments & Powers (AJMP)** | | |
| Barefoot Ankle Dorsi Flexion-Plantar Flexion Moment | BfAnklDfPfMom | Nm/kg |
| Unaltered Shoe Ankle Dorsi Flexion-Plantar Flexion Moment | UasAnklDfPfMom | Nm/kg |
| Altered Shoe Ankle Dorsi Flexion-Plantar Flexion Moment | AsAnklDfPfMom | Nm/kg |
| Barefoot Ankle Inversion-Eversion Moment | BfAnklInvEvrMom | Nm/kg |
| Unaltered Shoe Ankle Inversion-Eversion Moment | UasAnklInvEvrMom | Nm/kg |
| Altered Shoe Ankle Inversion-Eversion Moment | AsAnklInvEvrMom | Nm/kg |
| Barefoot Ankle Internal-External Rotation Moment | BfAnklIntExtlRMom | Nm/kg |
| Unaltered Shoe Ankle Internal-External Rotation Moment | UasAnklIntExtlRMom | Nm/kg |
| Altered Shoe Ankle Internal-External Rotation Moment | AsAnklIntExtlRMom | Nm/kg |
| Barefoot Ankle Power | BfAnklPwr | W/kg |
| Unaltered Shoe Ankle Power | UasAnklPwr | W/kg |
| Altered Shoe Ankle Power | AsAnklPwr | W/kg |
| **Knee Joint Moments & Powers (KJMP)** | | |
| Barefoot Knee Flexion-Extension Moment | BfKneeFlexExtnMom | Nm/kg |
| Unaltered Shoe Knee Flexion-Extension Moment | UasKneeFlexExtnMom | Nm/kg |
| Altered Shoe Knee Flexion-Extension Moment | AsKneeFlexExtnMom | Nm/kg |
| Barefoot Knee Adduction-Abduction Moment | BfKneeAddAbdMom | Nm/kg |
| Unaltered Shoe Knee Adduction-Abduction Moment | UasKneeAddAbdMom | Nm/kg |
| Altered Shoe Knee Adduction-Abduction Moment | AsKneeAddAbdMom | Nm/kg |
| BarefootKnee Internal-External Rotation Moment | BfKneeIntExtlRMom | Nm/kg |
| Unaltered Shoe Knee Internal-External Rotation Moment | UasKneeIntExtlRMom | Nm/kg |
| Altered Shoe Knee Internal-External Rotation Moment | AsKneeIntExtlRMom | Nm/kg |
| Barefoot Knee Power | BfKneePwr | W/kg |
| Unaltered Shoe Knee Power | UasKneePwr | W/kg |
| Altered Shoe Knee Power | AsKneePwr | W/kg |
| **Torsional Stiffness (TS)** | | |
| Unaltered Shoe Inversion Torsional Stiffness | UasInvTS | Nm/° |
| Altered Shoe Inversion Torsional Stiffness | AsInvTS | Nm/° |
| Unaltered Shoe Eversion Torsional Stiffness | UasEvrTS | Nm/° |
| Altered Shoe Eversion Torsional Stiffness | AsEvrTS | Nm/° |

**Note:** Abbreviation: BW, body weight; BH, body height; %GC, percentage of gait cycle; Nm/kg, Newton-meter per kilogram; W/kg, watts per kilogram; Nm/°, Newton-meter per degree.
